# An Electronic Analog of Synthetic Genetic Networks

**DOI:** 10.1371/journal.pone.0023286

**Published:** 2011-08-04

**Authors:** Edward H. Hellen, Evgenii Volkov, Jurgen Kurths, Syamal Kumar Dana

**Affiliations:** 1 Department of Physics and Astronomy, University of North Carolina Greensboro, Greensboro, North Carolina, United States of America; 2 Department of Theoretical Physics, Lebedev Physical Institute, Moscow, Russia; 3 Potsdam Institute for Climate Impact Research, Potsdam, Germany; 4 Indian Institute of Chemical Biology, Council of Scientific and Industrial Research, Kolkata, India; University of Maribor, Slovenia

## Abstract

An electronic analog of a synthetic genetic network known as the *repressilator* is proposed. The *repressilator* is a synthetic biological clock consisting of a cyclic inhibitory network of three negative regulatory genes which produces oscillations in the expressed protein concentrations. Compared to previous circuit analogs of the *repressilator*, the circuit here takes into account more accurately the kinetics of gene expression, inhibition, and protein degradation. A good agreement between circuit measurements and numerical prediction is observed. The circuit allows for easy control of the kinetic parameters thereby aiding investigations of large varieties of potential dynamics.

## Introduction

The concept of synthesizing simple gene units to realize a desired function or to reproduce a known function is new [1] in biological systems. After confirmation of the unit's desired functional behavior, a large assembly of such units can be organized to perform complex biological functions [2]–[4]. This is like engineering small integrated chips to build a computer to derive a targeted function. In efforts towards engineering biological functions, a *repressilator* was first demonstrated as a synthetic genetic clock expressed in *Escherchia coli* [5] producing oscillations in expressed protein concentrations. A mathematical model based on standard chemical kinetics was also proposed that predicted the observed oscillations.

The dynamics of coupled synthetic genetic networks (SGNs) was also investigated [6]–[7] theoretically to understand the generation of synchronous rhythm in an assembly of *repressilator*s via quorum sensing type interaction. Quorum sensing [8] is a form of exchanging information that a bacterial colony uses to develop a common rhythm. This quorum sensing type of indirect coupling is set-up between the SGN cells through diffusion of auto-inducing small molecules in a common medium. When the feedback via auto-inducing agents inside a SGN cell is reinforcing [7], the coupled dynamics show a state of in-phase synchrony, whereas when the feedback is repulsive, then the coupled dynamics show various possible states [9]–[11]: in-phase and anti-phase synchrony, inhomogeneous limit cycles, inhomogeneous steady states, and homogeneous steady states. Recently, in a biological experiment [12], evidence of in-phase synchronized quorum of genetic clock units was found. However, more complex features, as chaos, antiphase, and multistability in synchronous rhythm of coupled genetic clocks are yet to be observed experimentally.

Mathematical models are always a very useful tool to predict complex behaviors of dynamical systems using numerical simulations. Experimental verification of rich multistability requires an accurate knowledge of the model parameters which is often very challenging in biological experiments. An alternative experimental approach using electronic analogs of the SGN was undertaken [13]–[15] to confirm the numerical results and to search for possible coupled dynamics. Although it is difficult to simulate the biological experiment exactly in a circuit, the advantage of an electronic SGN is accessibility of system parameters and their controllability that allows a systematic exploration of known and predictable dynamics. Earlier [13]–[15] electronic circuit analogs of SGN displayed oscillations with 120° phase shifts between the oscillating variables, qualitatively in agreement with the genetic *repressilator*, however, the multistability of coupled SGNs was missing since access to and control of the system parameters was lacking.

In this paper, an electronic analog of the SGN is specifically designed to derive more accurate kinetic parameters of the *repressilator*. The goal is to control the parameters and thereby to realize desired sets of various kinetic parameters used in the simulations of the mathematical model. As a result, the circuit shows agreement between the measurements and the numerical predictions. The building block for the electronic *repressilator* is a circuit model for a single negative regulatory gene. This circuit shall be useful in a variety of other SGN investigations in addition to the *repressilator*. Designing electronic circuits of SGN also has the purpose of reverse engineering where knowledge of the biological experiments can be utilized for new technology and applications [16].

## Methods

### Genetic Network Repressilator

The structure of the *repressilator* consists of three repressive genes connected in a loop [5], with each gene producing repressor to the subsequent gene. The genes (*i* = 1,2,3) each produce their own mRNA, which translate the repressor protein. Gene 1′s repressor inhibits transcription of gene 2′s mRNA, 2′s repressor inhibits 3′s mRNA, and 3′s repressor inhibits 1′s mRNA. Taking into account standard chemical kinetics for production, degradation and inhibition, a dynamical system model of 6 first order differential equations was used for the mRNA and the protein concentrations [5].

An electronic analog of the *repressilator* was also proposed earlier [14]–[15], where they used three voltages as the variables, thus reducing the above model to a set of three differential equations. However, these circuits did not simulate the kinetic parameters of the *repressilator* model. A reduced three variable model is also used here but special care is taken to retain the parameters for the chemical kinetics as in the original model [5]. The reduced genetic network (RGN) *repressilator* is defined as,
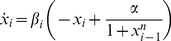
(1)where *i* = 1, 2, 3 for three genes, and the loop is closed by the condition *x*_0_ = *x*_3_. The product of the *i^th^* gene is *x*_i_, and its production is inhibited by *x_i-_*_1_. The parameter *α* accounts for the maximum transcription rate in the absence of an inhibitor, *β* is the decay rate of protein degradation and *n* is the Hill coefficient for inhibition. The Hill function is commonly used to account for sigmoidal binding kinetics. In this RGN model, there is no distinction between the mRNA and the transcribed repressor protein. We find that by this reduction, the fundamental features of the SGN model are not affected.

### Description of the RGN Circuit

**Figure 1:**
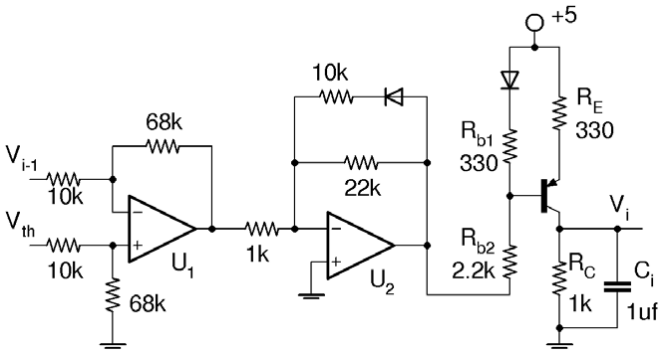
Electronic circuit analog of negative regulatory gene. *V_i_*_-1_ at the input is the concentration of inhibitory repressor. The circuit output *V*_i_ is the concentration of the gene's product. *V*_th_ accounts for the binding constant of the repressor to the gene's DNA. As inhibitor concentration *V*_i-1_ increases past *V*_th_, the voltage at the transistor base rises, turning the transistor's collector current off, thereby stopping gene production. Op-amps are LF412, diodes are 1N4148, *pnp* transistor is 2N3906, and +/−5 V supply.

The building block for the RGN *repressilator* circuit is the circuit for a single negative regulatory gene shown in Fig. 1. The desired dynamics are given by (1), where *x_i_* corresponds to the output of the circuit and *x_i_*_-1_ is the input. The goal is to use a circuit which accounts for the kinetics of gene inhibition, production of repressor, and degradation of repressor. The gene's product is analogous to the charge coming from the collector of the transistor and the rate of production is the transistor current. The concentration of product *x_i_* is proportional to the voltage *V_i_* across the capacitor, and the rate of decay is the current through *R_C_*. The kinetics of gene inhibition is determined by the circuitry that couples the input voltage *V_i_*_-1_ (the inhibitor) to the voltage at the base of the transistor.

#### Circuit Analysis

Here the circuit parameters are determined that correspond to particular values of kinetic parameters *α*, *β_i_*, and *n* in (1). The dynamical equation for voltage *V_i_* is
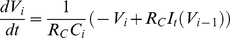
(2)where *I_t_*(*V_i_*_-1_) is the transistor's collector current and *V_i_*_-1_ is the variable input voltage. The equations are expressed in dimensionless form using dimensionless time *t*/*τ* where the time-scale is chosen by *τ*  =  *R_C_C*_0_. The capacitor value is then *C*_i_  =  *C*_0_/*β_i_* and (2) becomes
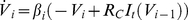
(3)where the dot denotes time derivative in dimensionless time *τ*. In order for the circuit to model the gene kinetics it is desired that *I_t_*(*V_i_*_-1_) approximates the Hill function,
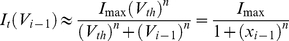
(4)where *x_i_*_-1_ =  *V_i_*_-1_/*V_th_* and *V_th_* represents an equilibrium constant for binding of repressor *x_i_*_-1_ to the gene's DNA. *I*_max_ is the maximum current through the transistor corresponding to gene transcription in the absence of an inhibitor. The production is half-maximal, *I_t_*  =  *I*_max_/2, when *V_i_*_-1_  =  *V_th_*. Dividing both sides of (3) by *V_th_*, the *α* and *β_i_* are given by *α*  =  *I*_max_ *R_C_*/*V_th_* and *β_i_*  =  *C*_0_/*C_i_*. Note that *I*_max_ is not due to saturation of the transistor since the voltage *I*_max_ *R_C_* is chosen so that the emitter-collector voltage across the transistor does not reach zero. Instead *I*_max_ is due to saturation of the op-amp as discussed below.

**Figure 2:**
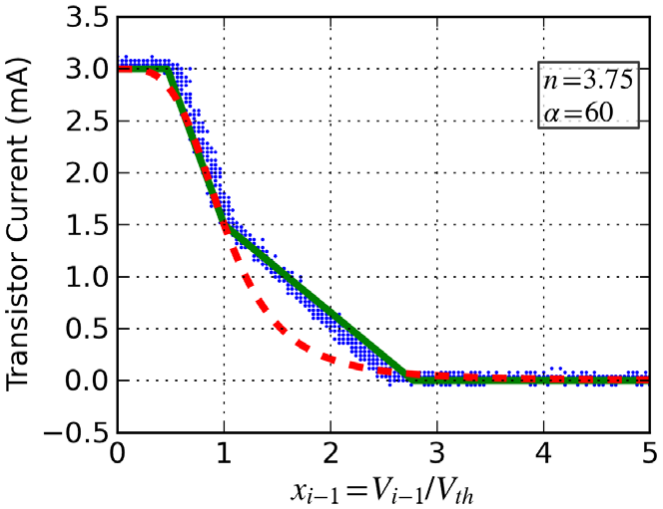
Hill function inhibitory response. Hill function inhibition of gene expression is approximated by the transistor current's dependence on input voltage *V*_i-1_ for the circuit in Fig. 1. Hill function (red dashed line), predicted current (green solid line), and measured current (blue dots). Maximum transistor current of 3 mA corresponds to maximum transcription rate α = 60. The Hill coefficient is *n* = 3.75.



Next we determine how the Hill coefficient *n* relates to the circuit parameters. The Hill function behavior consists of a transition of *V_i_* from one value to another one as *V_i_*_-1_ increases, with the transition occurring in the region around *V_i_*_-1_  =  *V_th_* as shown in Fig. 2. In addition the slope in the transition region is not constant. The circuit using the two op-amps U_1_ and U_2_ approximates this behavior by saturating the op-amp output and by using different gains in the transition region, a larger gain for *V_i_*_-1_ < *V_th_* and smaller for *V_i_*_-1_ > *V_th_*. Op-amp U_1_ is configured as a subtraction amplifier with output *G*_1_(*V*_i-1_ − *V*_th_)  =  *G*_1_Δ*V* where *G*_1_ is negative. Op-amp U_2_ has different gains *G*_+2_ and *G*_-2_ depending on the sign of Δ*V*. Taking saturation of the outputs into account gives the voltage at the output of U_2_,

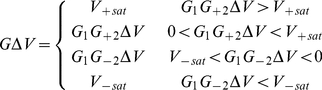
(5)where *V_±sat_* are the saturation levels.

The next step is to consider how *G*Δ*V* controls the transistor current. The voltage drop across *R*_b1_ is
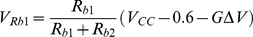
(6)where the forward bias voltage drop across the diode is 0.6 V and *V_CC_* is the supply voltage, +5 V. The diode in series with *R*_b1_ compensates for the transistor's emitter-base voltage drop, so that the voltage across *R*_b1_ is approximately the same as the voltage across *R*_E_. The current *I_t_*(*V*_i-1_) through *R*_E_ and the transistor is therefore (6) divided by *R*_E_. The maximum current *I*_max_ occurs when the output of U_2_ is saturated at *G*Δ*V*  =  *V_-sat_*, giving
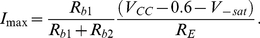
(7)


For comparison with the Hill function it is useful to express *I_t_* in terms of *I_max_* and the normalized input voltage *x*_i-1_,
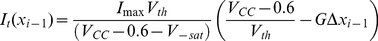
(8)where Δ*x_i_*_-1_  =  (*x*_i-1_−1).

In order to approximate the Hill function the overall gain *G*_1_ *G*_-2_ for *V*_i-1_<*V_th_* is chosen such that slope *dI_t_*/*dx*_i-1_ of the transistor current matches the slope of the Hill function at *x*_i-1_ =  1 (*V*_i-1_ = *V*_th_). At *x*_i-1_ = 1, the output *G*Δ*V* is not saturated, so *G*  =  *G*_1_ *G*_-2_. Equating the slopes gives the condition relating Hill coefficient *n* to the overall gain *G*_1_ *G*_-2_,
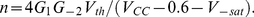
(9)


Choosing the gain *G*_+2_ (for *V*_i-1_>*V_th_*) to be less than *G*_-2_ improves the transistor current's approximation to the Hill function. We find that choosing *G*_+2_ ≅0.3*G*_-2_ works well for a range of parameter values *α*, *β_i_*, and *n*. Fig. 2 shows the Hill function (red dashed) and the predicted (green solid) and measured (blue dots) transistor current for *n* = 3.75.

#### Model Parameters, Circuit Parameters, and Design Considerations

Given a circuit it is useful to be able to easily determine the corresponding model parameters. From the previous section *α*, *β_i_*, and *n*, are expressed in terms of circuit parameters by
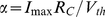
(10)

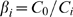
(11)

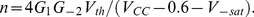
(12)


As an example, in Fig. 1 the gain for op-amp U_1_ is *G*_1_ = −6.8 and gain for op-amp U_2_ is *G*_-2_ =  −22 for non-saturated overall gain *G*_1_ *G*_-2_ = 150. For *V*_i-1_ > *V*_th_ the gain *G*_+2_ is approximately −6.9. For *V*_th_ = 50 mV, *C*_0_ = 1 µf, *I*_max_ = 3 mA, supply *V_CC_* = 5 V, and LF412 op-amp saturation *V_−sat_* = −3.5 V, the resulting model parameters are *α* = 60, *β_i_* =  1, and *n* = 3.8.

It is also useful to be able to determine circuit parameters that achieve a desired set of model parameters. Starting with (10), *I*_max_ *R_C_* must be far enough below the supply *V*_CC_ so that the emitter-collector voltage of the transistor never reaches zero. For *V*_CC_ = 5 V, *I*_max_ *R_C_* is chosen to be around 3 volts and the emitter-collector voltage never gets less than about 1volt so that the transistor never goes into saturation. The choice of *I*_max_ has some freedom. For *I*_max_ =  3 mA, this determines *R_C_* = 1 kΩ and *R_E_* = 330 Ω in order to get voltage drops of 1, 1, and 3 volts across *R_E_*, the transistor, and *R_C_*, respectively, when *I*_t_  =  *I*_max_. The remaining free circuit parameter to adjust for a desired value of *α* is *V*_th_. Rearranging (10) gives
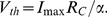
(13)


For example, for *I*_max_ *R_C_* = 3 V, a value of *α*  = 100 is obtained using *V*_th_ ≅30 mV. The capacitor value *C*_i_ to use for a desired *β_i_* is easily given by (11) as
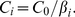
(14)


The third circuit parameter is the overall gain *G*_1_G_-2_ which is determined by *n* and *α*. Rearranging (12) gives
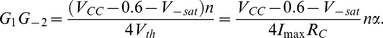
(15)


For *V_CC_* = 5 V and *V_−sat_* = −3.5 V, then *V_CC_* – 0.6− *V_−sat_* = 7.9 V, and with *I*_max_ *R_C_* = 3 V, then *G*_1_ *G*_-2_≈2*nα*/3. For example, if the desired parameter values are *α*  = 100 and *n* = 4, then the required overall gain is *G*_1_ *G*_-2_≈267 which can be split as desired between *G*_1_ and *G*_-2_. Thus, the circuit parameters that are adjusted to obtain a desired set of model parameters are *V_th_*, *C*_i_, and *G*_1_ *G*_-2_.

**Figure 3:**
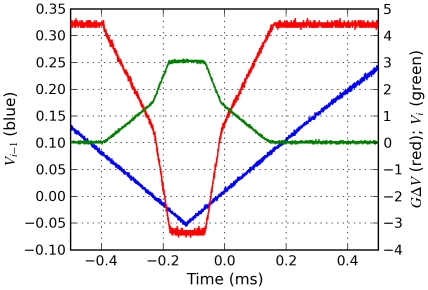
Measured time response of single gene circuit. Time response for the single gene circuit in Fig. 1 with *V*_th_ = 0 and *C*_i_ = 0. Input *V*_i-1_ (blue), op-amp output *GΔ*V (red), final output *V*_i_ (green). The red curve shows saturation of the op-amp output at +4.5 and –3.5 V when using a +/–5 V supply. The change in slope when *V*_i-1_  =  *V*_th_ is also apparent.

A careful selection of the op-amp is important for good approximation of the Hill function by the transistor current. The op-amp must be able to recover satisfactorily from saturation of its output. The circuit in Fig. 1 is tested with *V*_th_ set to zero and with *C_i_* = 0. Results for the LF412 are shown in Fig. 3. The input voltage *V*_i-1_ (blue line) is a triangle wave from a signal generator and the measured outputs are *G*Δ*V* (red line) from the output of U_2_ and the final output voltage *V*_i_ (green line). The red curve shows that the LF412 saturates at *V_+sat_*  =  +4.5 V and at *V_-sat_*  =  −3.5 V when using a ±5 V supply, and that the circuit makes the transition from high gain to low gain when *V*_i-1_ goes from negative to positive corresponding to *x_i_*_-1_ surpassing one. The green line shows the expected inhibitory response with respect to input *V*_i-1_, and the maximum value of *I*_max_ *R*_C_ = 3 V.

#### *Repressilator* Circuit

**Figure 4:**
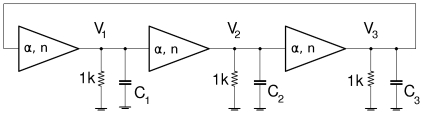
Electronic repressilator. Repressilator circuit constructed from a loop of three of the negative regulatory gene circuits of Fig. 1.

The electronic *repressilator* circuit consists of three negative regulatory gene circuits (Fig. 1) connected in an inhibitory loop as shown in Fig. 4. The triangle symbol contains the 2 op-amps, transistor, and circuitry which determine parameters *α* and *n*.

## Results and Discussion

The model parameters *α*, *β_i_*, and *n* are now ably determined by circuit parameters. Our results of circuit measurements and numerical predictions are shown for three cases: (1) identical genes, *β*-ratio  = 1∶1∶1; (2) gene *i* = 1 with faster decay, *β*-ratio  = 3∶1∶1; (3) gene *i*  = 1 with slower decay, *β*-ratio  = 0.3∶1∶1. Gene products are the normalized voltages *x*_1_ (blue), *x*_2_ (red), and *x*_3_ (green). Circuit measurements are solid lines, numerical predictions are dashed lines. The circuit parameters *V*_th_ and overall gain *G*_1_ *G*_-2_ were varied using (13) and (15) in order to set *α* and *n*. *V_th_* varied from 30 mV to 120 mV, and *G*_1_ *G*_-2_ from 73 to 220 corresponding to *α* = 25 to 100 and *n* = 3.0 to 6.6. The figures show results for (*α, n*)  =  (50, 6.6) and (100, 3.3). Other sets of parameters produced results with similar agreement of measurements and numerical predictions. The time constant was *τ*  =  *R_C_C*_0_  =  (1 kΩ)(1 μf)  = 1 ms. It follows that *C*_i_ =  (1 µf)/*β_i_*.

**Figure 5:**
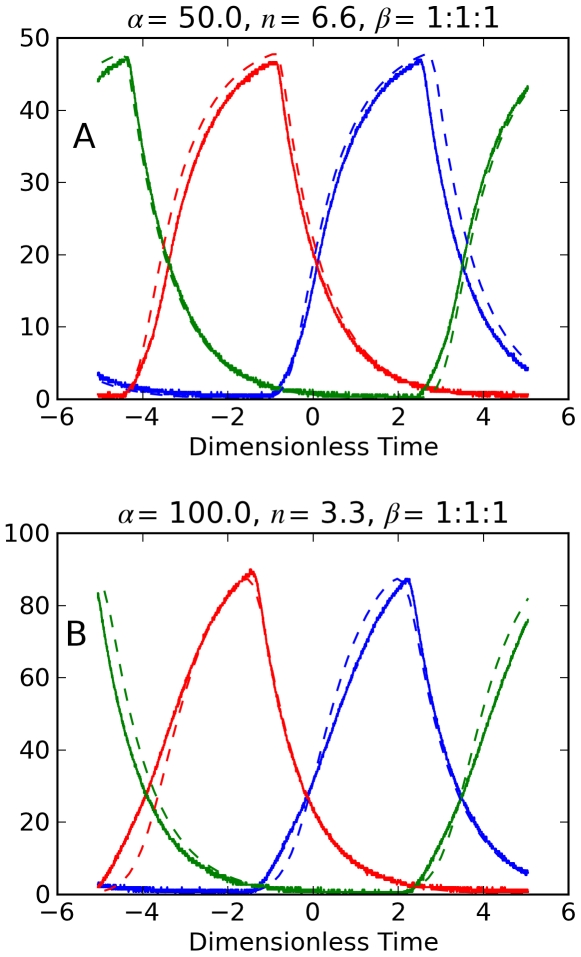
Electronic repressilator dynamics. Normalized voltages *x_i_* measured from RGN repressilator circuit (solid) and numerical predictions (dashed). *β*-ratio  = 1∶1∶1. Panel A (*α* = 50, *n* = 6.6); Panel B (*α* = 100, *n* = 3.3).

Fig. 5 shows the *repressilator* dynamics for *β*-ratio = 1∶1∶1 where *C*_i_ = 1 µf for each capacitor. The three state variables have the same shape, but with 120° phase shift. Numerical predictions are in close agreement with the measurements.

**Figure 6:**
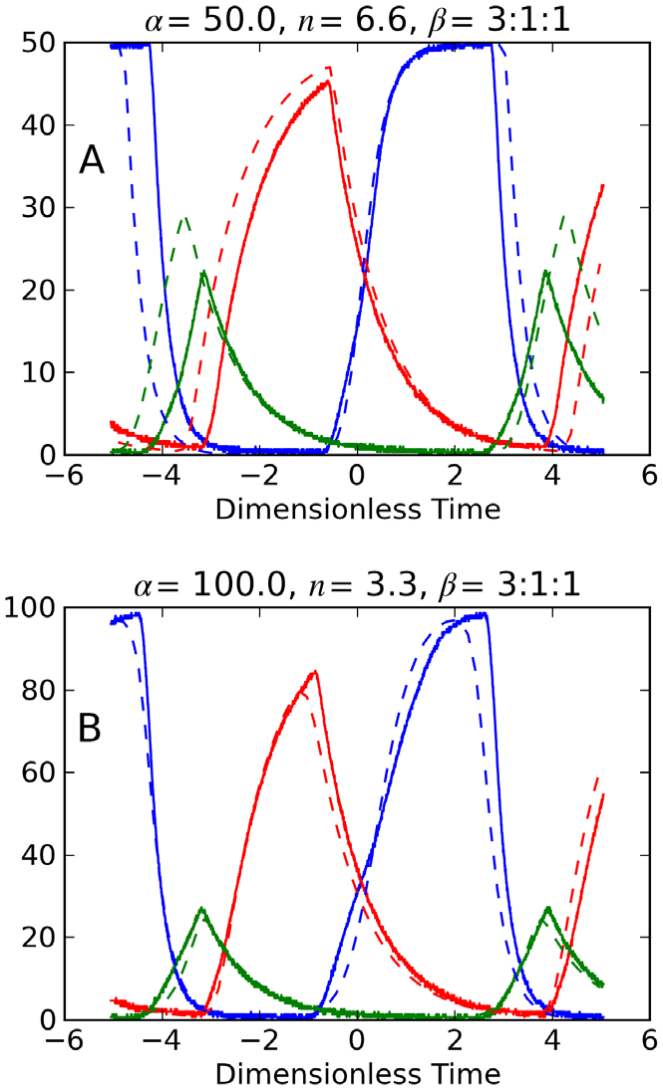
Electronic repressilator dynamics with one fast decay rate. Normalized voltages *x_i_* measured from RGN repressilator circuit (solid) and numerical predictions (dashed). *β*-ratio  = 3∶1∶1. Panel A (*α* = 50, *n* = 6.6); Panel B (*α* = 100, *n* = 3.3).

For the dynamics in Fig. 6 gene *i* = 1 has its capacitor reduced to 0.33 µf, so it has *β*_1_ = 3. Thus the *β* -ratio for the genes in the *repressilator* circuit is 3∶1∶1. Increasing the gene product's decay rate causes larger oscillations for the product, reduced oscillations for the gene's inhibitor, and an increased oscillation frequency for the *repressilator*.

**Figure 7:**
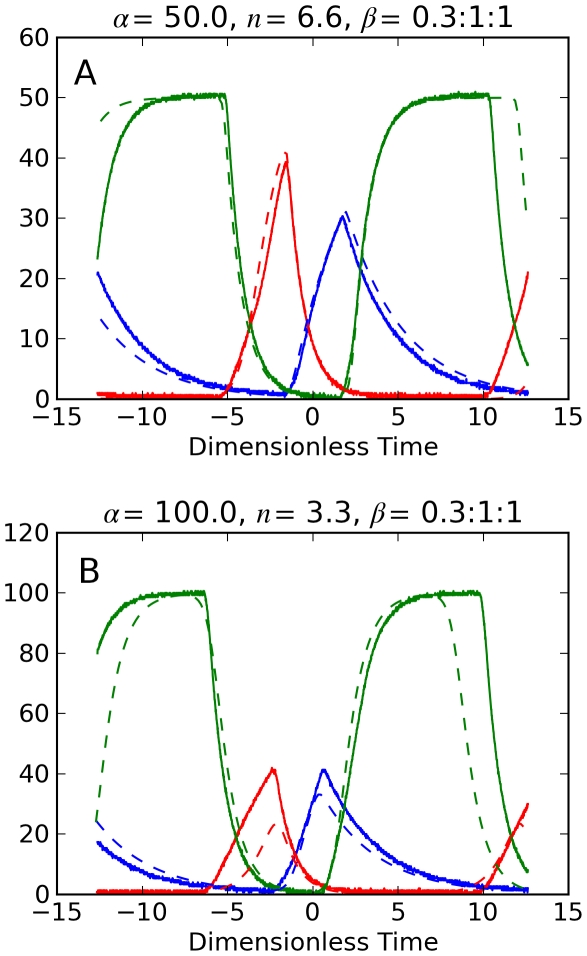
Electronic repressilator dynamics with one slow decay rate. Normalized voltages *x_i_* measured from RGN repressilator circuit (solid) and numerical predictions (dashed). *β*-ratio  = 0.3∶1∶1. Panel A (*α* = 50, *n* = 6.6); Panel B (*α* = 100, *n* = 3.3).

For the dynamics in Fig. 7 electronic gene *i* = 1 has its capacitor increased to 3.3 μf, so it has *β*_1_ = 0.3. Thus the *β*-ratio for the genes in the *repressilator* circuit is 0.3∶1∶1. Gene *i* = 1 now has reduced oscillations, its inhibitor gene *i* = 3 has increased oscillations, and the *repressilator* frequency has decreased.

The circuit presented here as an electronic analog of a synthetic genetic network known as the *repressilator* shows good agreement between experimental measurements and numerical predictions. The circuit includes control of parameters for the Hill function which is used to model the kinetics of gene expression and inhibition in the cyclic 3-gene network. Previous electronic analogs of the *repressilator* [14]–[15] did not concentrate on the kinetics and control of the parameters, and thereby did not capture many complex dynamical features. With the ability to control the model parameters, this circuit will be useful for investigations of multistability of coupled *repressilators* as well as for other SGN dynamics.
